# Best predictive single nephrometry score component to correlate with achievement of trifecta outcome in laparoscopic and robotic surgery

**DOI:** 10.1186/s12894-024-01518-4

**Published:** 2024-06-28

**Authors:** Sappaya Suppanuntaroek, Kyle Garcia, Christopher Combates, Carly Deal, Irasema Concepción Paster, Christian C. Morrill, Ken Batai, Benjamin Lee

**Affiliations:** 1 Department of Urology, University of Arizona, Tucson, AZ USA; 2 Urology, Queen Savang Vadhana Memorial Hospital, Chonburi, Thailand; 3 School of Osteopathic Medicine, A. T. Still University, Mesa, AZ USA; 4 Roswell Park Cancer Institute, Cancer Prevention and Control, Buffalo, NY USA

**Keywords:** Renal cell carcinoma, Robotic partial nephrectomy, Laparoscopic partial nephrectomy, Nephrometry system, Trifecta outcomes

## Abstract

**Background:**

To evaluate the predictive value of individual components of the R.E.N.A.L scoring system for Laparoscopic (LPN) and Robotic Partial Nephrectomy (RPN).

**Methods:**

Patients that had undergone a Laparoscopic (LPN) or Robotic Partial Nephrectomy (RPN) between 2018 and 2023 were reviewed. Our data collection included Race, Ethnicity, Age, BMI, R.E.N.A.L nephrometry score, and complications. Cases that achieved trifecta outcomes were designated as “Group A” and cases that did not achieve trifecta were “Group B”. All the data were collected using REDCap database.

**Results:**

A total of 111 cases were included, Group A consisted of 82% of all cases, whereas Group B 18%. Radius score demonstrated significant distinction concerning trifecta attainment and was the most predictive component of the 5 scoring metrics of the nephrometry system. In a subgroup analysis, R-score of 3 or a renal mass measuring ≥ 7 cm, was a significant independent negative predictor for trifecta outcomes, as well as tumor size at presentation.

**Conclusion:**

Renal nephrometry score is predictive of trifecta outcomes for patients undergoing laparoscopic or robotic partial nephrectomy. Radius of mass was the most effective predictive component of the nephrometry score for trifecta prediction.

## Background

Nephron-sparring surgery (NSS) or Partial nephrectomy (PN) is the preferred treatment for Stage T1a Renal Cell Carcinoma (RCC), with growing experience in T1b renal masses with select T2 renal masses eligible with favorable anatomy [1]. A considerably complex operation, PN requires significant skill to achieve optimal oncologic outcomes, minimize post-operative complications and maintain quality of life. The R.E.N.A.L. nephrometry score is an established anatomical system for categorizing complexity of renal tumors prior to surgery. Multiple studies have shown Nephrometry scores strongly correlate with effective PN [2–5] and predicts the attaining of “trifecta outcome” (reduced global renal ischemia, minimal complications, and high rates of negative margins) [4, 6]. Herein, we evaluate the predictive value of individual components of the R.E.N.A.L scoring system for Laparoscopic (LPN) and Robotic Partial Nephrectomy (RPN).

## Methods

**Fig. 1:**
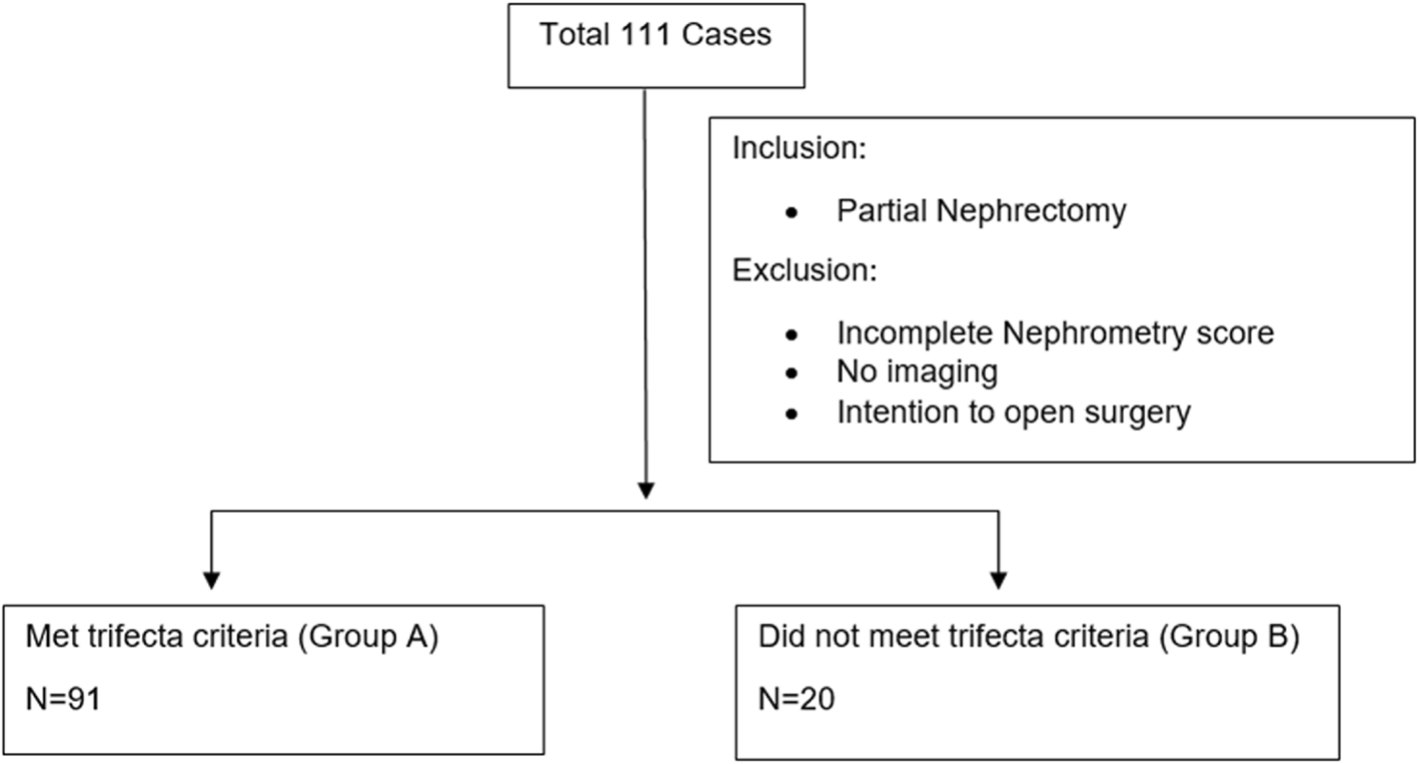
Group assignment

An institutional, IRB approved, database was reviewed for LPN and RPN cases performed at a single, high volume, academic center between 2018 to 2023. All cases were reviewed against the criteria for R.E.N.A.L. nephrometry scoring. Cases with no available imaging or incomplete data were excluded. Figure 1 shows group assignment. Pre-operative imaging (Computed Tomography, CT, and/or Magnetic Resonance Imaging, MRI) were reviewed by independent researchers and scored according to accepted R.E.N.A.L nephrometry parameters.

Demographic data was gathered and the R.E.N.A.L score was calculated for each partial nephrectomy. The R.E.N.A.L. score is calculated using the radius of the renal mass (R), the exophytic/endophytic location of the mass relative to the renal parenchyma (E), nearness of the mass to the collecting system (N), anterior/posterior location of the mass (A), and location of the mass relative to polar lines (L) [7].  All cases were stratified as low (4-6) intermediate (7-9) or high complexity (10-12).  We also determined whether each case achieved Trifecta outcomes. The goals of trifecta outcome vary slightly in the literature [8–10], but for the purposes of this study, the definition of Trifecta goals and outcomes were warm ischemia time <25 minutes, negative margin, and no Clavian complications >3 which, in the case of partial nephrectomy include renal hemorrhage requiring surgical exploration or intervention, urine leak, and/or kidney loss. Minor complications were also tracked in accordance with the Clavien-Dindo system [11]. All the data were collected using the REDCap database.

Data were analyzed using IBM SPSS Statistical Software (version 29.0.0). All categorical variables were presented as number of occurrences and percentages of the whole. The continuous variables were represented with median and interquartile range (IQR) or mean and standard deviation (SD) as appropriate. The linear correlation between variables was identified using Spearman’s or Pearson’s Correlation Coefficients. Categorical variables were compared using Chi-square. Continuous variables were compared using ANOVA and K-independent non-parametric test. The specific differences between groups were performed with multivariable logistic regression models. Significant levels were defined as *P* < 0.05.

## Results

A total of 111 cases were included in the study. The demographic data of the study cohort are listed in Table 1. Median age was 62 years old (54–71). The median BMI, R.E.N.A.L score, average warm ischemic time were 29.0 (24.3–34.5), 7.00 (6–9), and 17.0 min (15.0–21.0), respectively. Trifecta outcomes were achieved in 91 cases (82%) (< 25-minute warm ischemia time, negative surgical margins, and no high-grade complications). We designated cases that achieved trifecta outcomes as “Group A” (82%) and those that did not as “Group B” (18%).

**Table 1:** Characteristics of patients included in the study

	Total	Trifecta Met	Trifecta Not Met	*P*
n (%)	111 (100.0)	91 (82.0)	20 (18.0)	
Warm ischemia time (min.), median (IQR)	17.0 (15.0–21.0)	17.0 (15.0–19.0)	26.0 (13.5–29.0)	0.03
Warm ischemia time, n (%)				< 0.001
<25 min	98 (88.3)	91 (100)	7 (35.0)	
≥ 25 min	13 (11.7)	0 (0.00)	13 (65.0)	
Surgical margin, n (%)				< 0.001
Negative, n (%)	104 (94.9)	91 (100)	13 (61.1)	
Positive, n (%)	7 (6.3)	0 (0.00)	7 (38.9)	
Any surgical complication, n (%)				0.36
No	90 (81.1)	75 (82.4)	15 (75.0)	
Yes	21 (18.9)	16 (17.6)	5 (25.0)	
Age at surgery, median (IQR)	62.0 (54.0–71.0)	63 (55.0–71.0)	57.5 (48.8–70.3)	0.15
Gender, n (%)				
Male	65 (58.6)	53 (58.2)	12 (60.0)	
Female	46 (41.4)	38 (41.8)	8 (40.0)	
Race and Ethnicity, n (%)				0.91
Non-Hispanic White	52 (46.8)	42 (46.2)	10 (50.0)	
Hispanic (any race)	38 (34.2)	32 (35.2)	6 (30.0)	
Other/Unknown/Mixed	21 (18.9)	17 (18.7)	4 (20.0)	
Body Mass Index, median (IQR)	29.0 (24.3–34.5)	28.8 (24.2–34.0)	31.8 (25.5–35.8)	0.53
Hypertension (Yes), n (%)	72 (64.9)	58 (63.7)	14 (70.0)	0.60
Diabetes (Yes), n (%)	32 (28.8)	29 (31.9)	3 (15.0)	0.13
Chronic Kidney Disease (Yes), n (%)	21 (18.9)	16 (17.6)	5 (25.0)	0.44
Smoking (Yes), n (%)	49 (44.1)	41 (45.1)	8(40.0)	0.68
Laterality, n (%)				0.89
Unilateral	96 (86.5)	68 (85.7)	18 (90.0)	
Bilateral	15 (13.5)	13 (14.3)	2 (10.0)	
Tumor size at presentation (cm), median (IQR)	3.0 (2.2–4.6)	2.8 (2.0–4.0)	4.6 (2.8–6.9)	0.05
Length of hospital stay (day), median (IQR)	2.0 (1–2)	2.0 (1–2)	2.0 (1–3)	0.07
Estimated blood loss (ml), median (IQR)	120.0 (50.0-242.5)	100.0 (50.0-200.0)	150.0 (100.0-250.0)	0.15
Pre-operative GFR (mL/min/1.73m^2^), median (IQR)	81.1 (58.6-114.5)	81.1 (58.0-108.9)	86.4 (58.9-139.6)	0.59
Post-operative GFR (mL/min/1.73m^2^), median (IQR)	81.3 (66.2–106.0)	81.3 (60.0-106.0)	81.3 (73.3–106.0)	0.72
R.E.N.A.L nephrometry score (median, IQR)	7.0 (6–9)	7.0 (6–9)	9.0 (6–11)	0.28
Nephrometry score, n (%)				0.08
Low (4–6)	24 (21.6)	16 (17.6)	8 (40.0)	
Intermediate (7–9)	49 (44.1)	43 (47.3)	6 (30.0)	
High (10–12)	38 (34.2)	32 (35.2)	6 (30.0)	
Radius, n (%)				0.02
≤4 (+ 1)	74 (66.7)	65 (71.4)	9 (45.0)	
>4 and < 7 (+ 2)	29 (26.1)	22 (24.2)	7 (35.0)	
≥7 (+ 3)	8 (7.2)	4 (4.4)	4 (20.0)	
Endophytic/Exophytic, n (%)				0.97
≥50% Exophytic (+ 1)	26 (23.4)	21 (23.1)	5 (25.0)	
<50% exophytic (+ 2)	61 (55.0)	50 (54.9)	11 (55.0)	
Entirely endophytic (+ 3)	24 (21.6)	20 (22.0)	4 (20.0)	
Nearness, n (%)				0.68
≥7 (+ 1)	31 (27.9)	27 (29.7)	4 (20.0)	
>4 and < 7 (+ 2)	19 (17.1)	15 (16.5)	4 (20.0)	
≤4 (+ 3)	61 (55.0)	49 (53.8)	12 (60.0)	
Anterior or Posterior, n (%)				0.57
Anterior	52 (46.8)	33 (36.3)	7 (35.0)	
Neither	19 (17.1)	14 (15.4)	5 (25.0)	
Posterior	40 (36.0)	44 (48.4)	8 (40.0)	
Location, n (%)				0.60
entirely above upper polar line or below lower polar line (+ 1)	43 (38.7)	37 (40.7)	6 (30.0)	
mass crosses polar line (+ 2)	28 (25.2)	23 (25.3)	5 (25.0)	
>50% of mass across polar line, entirely between polar line or mass crosses axial midline (+ 3)	40 (36.0)	31 (34.1)	9 (45.0)	
Pathological Stage, n (%)				0.08
pT1a	79 (71.2)	68 (74.7)	11 (55.0)	
pT1b	24 (21.6)	18 (19.8)	6 (30.0)	
pT2 and pT3	8 (7.2)	5 (5.5)	3 (15.0)	

Length of stay (LOS) and pre-operative glomerular filtration rate (GFR) did not exhibit statistically significant differences between Group B and Group A, with values of 2.0 days and 2.0 days (*p* = 0.07), and 81.1 ml/min/1.73 m² and 86.4 ml/min/1.73 m² (*p* = 0.59), respectively. Group B displayed higher Nephrometry scores (*p* = 0.28) and greater blood loss (*p* = 0.15) in comparison to Group A. Notably, the Radius score demonstrated a significant distinction (*p* = 0.02) concerning Trifecta attainment within all Nephrometry components and was the most predictive component of the 5 scoring metrics of the nephrometry system. No correlations were observed among the Trifecta outcome and gender, hypertension, diabetes, chronic kidney disease, smoking, age, BMI, or laterality.

In Table 2, a subgroup analysis was conducted to ascertain the predictive components of Trifecta outcomes. Notably, an R-score of 3 or a renal mass measuring ≥ 7 cm emerged as a significant independent negative predictor for Trifecta outcomes (*p* = 0.04, odds ratio = 5.66 (95% CI: 1.06–30.12)), including adjustment for age, gender, diabetes, and chronic kidney disease. Additionally, following adjustment of the same model, the tumor size at presentation was a negative predictor of trifecta outcome (*p* = 0.02, odds ratio = 1.36 (95% CI: 1.04–1.79)) (Table 3).

**Table 2:** R.E.N.A.L. Nephrometry Score (categorical) association with not meeting Trifecta

	Unadjusted Model		Adjusted Model 1		Adjusted Model 2		
	OR (95% CI)	*P*	OR (95% CI)	*P*	OR (95% CI)		*P*
Low	Reference		Reference		Reference		
Intermediate	0.74 (0.22–2.52)	0.64	0.71 (0.20–2.47)	0.59	0.57 (0.16–2.06)		0.39
High	2.67 (0.79-9.00)	0.11	2.40 (0.64-9.00)	0.20	2.33 (0.67–8.15)		0.19
Age	1.02 (0.95–1.09)	0.58	0.99 (0.95–1.03)	0.53			
Sex							
Female	Reference		Reference				
Male	0.93 (0.35–2.50)	0.89	1.11 (0.39–3.15)	0.84			
Diabetes							
No	Reference		Reference		Reference		
Yes	0.38 (0.10–1.39)	0.14	0.37 (0.09–1.46)	0.16	0.27 (0.06–1.30)		0.10
CKD							
No	Reference		Reference				
Yes	1.56 (0.50–4.92)	0.45	2.55 (0.71–9.21)	0.15			

Adjusted model 1 include everything on the table

Adjusted model 2 Include what was left in backward section

**Table 3:** Factors associated with not meeting Trifecta (adjusted by model1)

3	Unadjusted		Adjusted		
	OR (95% CI)	*P*	OR (95% CI)		*P*
R.E.N.A.L. Score (continuous)	1.20 (0.95–1.51)	0.14	1.14 (0.89–1.47)		0.30
Radius (ordinal)	2.58 (1.26–5.25)	0.009	2.36 (1.10–5.05)		0.03
Radius (categorical)					
≤4 (+ 1)	Reference		Reference		
>4 and < 7 (+ 2)	2.30 (0.77–6.90)	0.14	2.31 (0.74–7.17)		0.15
≥7 (+ 3)	7.22 (1.53–34.07)	0.01	5.66 (1.06–30.12)		0.04
Endophytic/Exophytic (ordinal)	0.92 (0.45–1.89)	0.81	0.88 (0.41–1.91)		0.75
Endophytic/Exophytic (categorical)					
≥50% Exophytic (+ 1)	Reference		Reference		
<50% exophytic (+ 2)	0.92 (0.29–2.99)	0.90	0.80 (0.22–2.86)		0.73
Entirely endophytic (+ 3)	0.84 (0.20–3.59)	0.81	0.78 (0.17–3.58)		0.75
Nearness (ordinal)	1.24 (0.70–2.23)	0.46	1.10 (0.60–2.04)		0.75
Nearness (categorical)					
≥7 (+ 1)	Reference		Reference		
>4 and < 7 (+ 2)	1.80 (0.39–8.25)	0.45	1.53 (0.32–7.38)		0.60
≤4 (+ 3)	1.65 (0.49–5.63)	0.42	1.29 (0.36–4.65)		0.70
Anterior or Posterior (categorical)			0.99 (0.57–1.75)		
Anterior	Reference		Reference		
Neither	1.96 (0.55–6.99)	0.30	2.23 (0.57–8.68)		0.25
Posterior	1.17 (0.38–3.54)	0.79	0.97 (0.30–3.18)		0.96
Location (ordinal)	1.34 (0.76–2.36)	0.31	1.23 (0.67–2.26)		0.51
Location (categorical)					
entirely above upper polar line or below lower polar line (+ 1)	Reference		Reference		
mass crosses polar line (+ 2)	1.34 (0.37–4.90)	0.66	1.09 (0.29–4.16)		0.90
>50% of mass across polar line, entirely between polar line or mass crosses axial midline (+ 3)	1.79 (0.57–5.59)	0.32	1.50 (0.45–5.03)		0.51
Pathological Stage (ordinal)	1.96 (0.97–3.97)	0.06	1.98 (0.94–4.13)		0.07
Pathological Stage (categorical)					
pT1a	Reference		Reference		
pT1b	2.06 (0.67–6.33)	0.21	2.18 (0.69–6.88)		0.19
pT2 and pT3	3.71 (0.77–17.77)	0.10	3.59 (0.68-19.00)		0.13
Tumor size at presentation (continuous)	1.37 (1.08–1.75)	0.01	1.36(1.04–1.79)		0.02

Adjusted model includes Age, Sex, Diabetes, and Chronic kidney disease

Table 4 shows there was only one major (high-grade) complication (Clavien Grade III), accounting for 0.9% of cases, which involved a pseudoaneurysm requiring embolization. Additionally, there were 11 (52.4%) Clavien-Dindo Grade I complications and 9 cases (46.7%) Clavien-Dindo Grade II complications.

**Table 4:** Complications

	Total	Trifecta Met	Trifecta Not Met
Complications, N(%)	21	16	5
Clavien I	13	10	3
Clavien II	7	6	1
Clavien III	1	0	1

## Discussion

Partial nephrectomy is the recommended treatment for stage I, and II renal cancer [12]. In 1993, laparoscopic partial nephrectomy was introduced and described as a reproducible technique by IS Gill et al. [13]. Laparoscopic surgery has been shown to lead to decrease in length of stay, analgesic used, and more rapid return to regular diet compared to open surgery [14]. In the past decade, robotic surgery has become more prevalent and associated with improved outcomes in patients with complex renal masses when compared to pure laparoscopic surgery [15], The Nephrometry scoring system was provided as an aiding tool for the comparison of renal masses in reproducible operation. Among various nephrometry scores, the R.E.N.A.L. is commonly used for determining the complexity and feasibility of surgery [7].

The trifecta criteria, as previously described, was established as a means of describing the success of a partial nephrectomy operation [16–19]. Campi et al. demonstrated that opinions differ on the precise definition of trifecta outcomes [20]. Most significantly, some studies evaluate actual decrease in renal function post-operatively in place of determining the success of an operation base on an arbitrary warm ischemia time [20, 21]. As previous studies have demonstrated that longer warm ischemia time is associated with higher rates of post-operative acute renal failure and new-onset Stage IV chronic kidney disease, we chose to use the generally accepted cut off of 25-minute warm ischemia time as the determinant of success for our study [22]. Also contributing to our decision to include warm ischemia in the definition of trifecta outcome is the fact that striving for a decreased warm ischemia time is a measurable goal towards which quantifiable improvements can be made. As a training institution, we encourage these quantifiable metrics in order to track the surgical improvement of residents and faculty and, thus, we have decided to include warm ischemia times as a part of our definition of trifecta outcomes.

Other studies have advocated for the off-clamp approach in select cases in an effort to increase renal function preservation [23, 24]. In an elegant 1:1 propensity score matching retrospective analysis, Simone et al. demonstrated no increase in positive surgical margin or severe complication rates in those patients who underwent off-clamp robotic partial nephrectomy compared to on-clamp [24]. However, they did demonstrate a higher rate of trifecta outcome in the off-clamp group [24]. Furthermore, they demonstrated that a warm ischemia time > 20 min was independently negatively associated with trifecta achievement [25].

In addition, multiple studies have analyzed the relationship between surgical approach and trifecta outcome [4, 8, 10, 26]. In a direct comparison between open and robotic PN, Campi et al. found that robotic PN was a significant predictor for achieving trifecta outcomes [27]. It has also been found that the radius score (R-score), location score (L-score), and the overall R.E.N.A.L score affect trifecta outcomes in previous studies articles [4, 9], but few studies have analyzed which of the 3 components have the best predictive value of achieving optimal outcomes.

In our study, the only component of the nephrometry score that was independently predictive of Trifecta outcomes was radius of the mass (R-score). There were a total of eight renal masses of radius greater than 7 cm ( stage T2) included in our study. Trifecta outcomes was achieved in only four of these cases (50%). By comparison, renal masses of radius between 4 and 7 cm (stage T1b) achieved trifecta outcomes in 75.8% of cases, and masses of radius less than 4 cm (stage T1a) achieved trifecta outcomes in 87.7% of cases. This is consistent with multiple previous studies. Carbonara et al. presented a multicenter analysis involving endophytic tumor partial nephrectomy, wherein the size of the renal mass was identified as the sole significant predictor for the achievement of Trifecta outcomes [28]. In 2012, W. Mayer investigated the correlation between the Nephrometry score and warm ischemia time [26]. In his study, he demonstrated that the R-score and N-score (proximity to the collecting system) were predictive of longer warm ischemia time and an increased likelihood of collecting system entry [26]. In our series, collecting system was opened > 50% of the cases, reflecting the need for closure to avoid fistula. Our fistula rate is < 1% overall. In addition to a more complicated surgery due to larger size and more difficult location of these masses, these results could also be attributable to a more advanced tumor histology that exists in larger lesions which could, in and of itself, complicate the operation [29]. R. H. Thompson identified 2,675 patients treated surgically for RCC between 1989 and 2007 [30]. The article reported that for each 1 cm increase in RCC tumor size, there was a corresponding 25% increase in the incidence of high-grade disease (Fuhrman grade 3–4) [30]. Furthermore, histology can contribute to surgical complexity; in particular, clear cell histology has been found to be associated with expansive pseudocapsular invasion and infiltrative pseudocapsular invasion [31].

The literature demonstrates other predictive factors such as location-score. M. Kang studied 362 cases following RAPN to identify predictors of Pentafecta outcome (Trifecta outcome plus GFR preservation of more than 90% and no stage upgrade of chronic kidney disease) in small renal tumors [32]. They reported that the significant predictors included preoperative GFR, hypertension, tumor size, L-score, and surgeon’s experience [32]. Additionally, M. Tsivian et al. studied the risk factors of conversion from partial to radical nephrectomy [33]. 168 cases were converted, and it was found that posterior, middle location (on anteroposterior axis), and hilar location of the tumor were associated with increased odds of conversion [33]. In addition, alternative scoring systems such as PADUA and C-Index scores have been evaluated. Our calculated odds ratio with regards to the predictive ability of the isolated R-score component of the Nephrometry score, while significant, was not as strong as the predictive ability of the PADUA nor the C-Index Score, as investigated by Ates et al. [4, 34, 35].

In our study, the patients in Group B were significantly younger than the patients in Group A. This could possibly be attributed to the more aggressive or complex tumors in younger patients with inherited tumor disorders. Sharma et al. investigated the effect of age on complications following robot partial nephrectomy and found that perioperative complications were significantly higher in more elderly patients (*p* = 0.041) but there was no statistically significant difference in *major* complications between the elderly and younger patient groups [36]. Enhanced attainment of trifecta outcomes in intricate tumors appears to be linked to the adeptness of the surgeon and the utilization of robotic surgical techniques [15, 37]. Given that our study is single institution, we did not stratify by surgeon or surgical experience.

Our study is limited due to the retrospective nature of our database and is therefore limited in its ability to draw clear associations. Our sample size is also relatively small, in particular the Group B subset of patients lacks the sample size for strong statistical power, as manifest by the wide confidence intervals. As a single institutional database, the external validity of our study may be limited by parameters unique to our institution, patient population, or surgical techniques. Future studies are needed, including studies with long-term post-operative follow up data, to further elucidate predictive factors of Trifecta outcome, as well as the effect of Trifecta outcomes on long-term patient’s wellness.

## Conclusion

We found that R.E.N.A.L Nephrometry score is useful for the prediction of achieving trifecta outcome (reduced global renal ischemia, no complications, and negative surgical margins) for patients who underwent Laparoscopic or robotic partial nephrectomy and we found radius of mass to be the most effective predictive component of the Nephrometry score. Thus, the Nephrometry score is a valuable tool for urologists when it comes to surgical planning.

## Data Availability

The dataset used and analyzed during the current study are available from the corresponding author on reasonable request.

## Abbreviations

NSS: Nephron-sparring surgery
PN: Partial nephrectomy
RCC: Renal cell carcinoma
R.E.N.A.L.: (R)-Radius of renal mass, (E)-Exophytic/endophytic location of the mass relative to the renal parenchyma, (N)-Nearness of the mass to the collecting system, (A)-Anterior/posterior location of the mass, (L)-location of the mass relative to polar lines
LPN: Laparoscopic partial nephrectomy
RPN: Robotic partial nephrectomy
IRB: Institutional Review Board
CT: Computed Tomography Scan
MRI: Magnetic Resonance Imaging
REDCap: Research Electronic Data Capture
IMB SPSS: International Business Machines Statistical Package For Social Sciences
IQR: Interquartile Range
SD: Standard Deviation
ANOVA: Analysis of Variance
BMI: Body Mass Index
LOS: Length of Stay
GFR: Glomerular Filtration Rate
R: Score-Radius Score
CI: Confidence Interval
L: Score-Location Score
CKD: Chronic Kidney Disease
RAPN: Robotic Assisted Partial Nephrectomy
